# Exercise During Pregnancy: Knowledge and Beliefs Among Females in Saudi Arabia

**DOI:** 10.7759/cureus.30672

**Published:** 2022-10-25

**Authors:** Abdulrahim M Gari, Sarah S Aldharman, Wedad O Alalawi, Ethar H Alhashmi Alamer, Aeshah A Alnashri, Fatimah A Bomouzah

**Affiliations:** 1 Department of Obstetrics and Gynecology, Faculty of Medicine, Umm Al-Qura University, Makkah, SAU; 2 College of Medicine, King Saud Bin Abdulaziz University for Health Sciences, Riyadh, SAU; 3 College of Medicine, Umm Al-Qura University, Makkah, SAU; 4 College of Medicine, King Faisal University, Al-Ahsa, SAU

**Keywords:** females, beliefs, knowledge, pregnancy, exercise

## Abstract

Background

Exercise during pregnancy helps to promote health benefits for both the mother and fetus. One of the concerns among Saudi pregnant women is physical inactivity. The objective of this study was to assess females' knowledge, beliefs, and practices toward exercise during pregnancy in Saudi Arabia. Also, we investigated the most common barriers to exercising during pregnancy.

Methods

The study was a cross-sectional study that included all Saudi females aged 18 years and above and excluded non-Saudi females and those aged less than 18 years. A self-administered survey was distributed on social media platforms. The collected data were coded and analyzed using SPSS version 23 (IBM Corp., Armonk, NY).

Results

A total of 1207 participants were enrolled in the study. In regards to general knowledge and awareness levels, 1002 (83%) participants had a high level of knowledge and awareness, and 205 (17%) had a low level of knowledge and awareness. Exercise during pregnancy is essential was agreed on by 853 (70.7%) participants. The most reported sources of information on prenatal exercise were found to be websites, as reported by 56.7% of the participants. About 42.4% of the participants were sometimes exercising during pregnancy. The most common type of antenatal exercise was found to be walking, as mentioned by 83.5% of the participants. The most commonly reported barrier to practicing antenatal exercises was found to be fatigue, as reported by 53.9% of the participants, and lack of time. Age was found to be significantly associated with the level of beliefs, awareness, and knowledge regarding antenatal care. A statistically significant association was found between marital status and level of beliefs, awareness, and knowledge regarding antenatal care with married participants tending to be having a higher level of knowledge and awareness compared to other groups. Occupation and level of beliefs, awareness, and knowledge regarding antenatal care were found to be significantly associated.

Conclusion

We found good general knowledge and awareness levels regarding exercise during pregnancy. Beliefs toward exercise during pregnancy were below average. Future studies on how to promote regular exercise during pregnancy are recommended.

## Introduction

The impact of physical activity during pregnancy is one of the significant issues among pregnant women [1]. Lack of exercise and excessive prenatal weight gain are two significant risk factors for obesity following pregnancy; thus, all women of reproductive age should begin regular exercise to support them throughout pregnancy and delivery [1]. Excessive prenatal weight gain is seen as a major rising concern among pregnant women in Saudi Arabia and throughout the world, and it is linked to feto-maternal issues such as gestational diabetes, macrocosmic baby birth, and preeclampsia [2-4]. Moreover, it has been estimated that around 16.4% of Saudi women were inactive, and 9.1% lacked awareness about physical activity's importance during pregnancy [5]. Consequently, appropriate education toward exercise will provide a positive attitude about their health during the gestational and postpartum periods. Healthcare providers play an important role in raising awareness and educating pregnant women about the importance of physical activity. On the other hand, the influence of social and cultural beliefs and family support are factors affecting pregnant women's thoughts toward physical activity [6]. In the past, pregnant women were advised against physical exercise since it was believed to be harmful to their health and may result in miscarriage, low birth weight, or early delivery. However, recent research has found that regular physical activity during pregnancy has several benefits for both the mother and the fetus [7-10]. Many recommendations encourage frequent, moderate-intensity exercise as an integral part of maternal preventive care [11-14]. However, due to the persistence of misconceptions and beliefs and the lack of knowledge, most pregnant women tend to lead a sedentary lifestyle devoid of physical activities, especially in the third trimester [15]. Females in Saudi Arabia exhibit a reduction in physical activity throughout pregnancy, with less than 50% maintaining a level of exercise that has a positive influence on their general health and birth outcomes [16]. According to the National Institute for Health and Care Excellence (NICE) guidelines and the American College of Obstetricians and Gynecologists (ACOG) guidelines, antenatal exercise has minimal risks and paramount benefits with some modifications that fit the mother and the fetus. The recommendation of an exercise program under supervision has shown a lot of benefits in managing weight during pregnancy and reducing the risk of gestational diabetes mellitus (GDM) [9]. A cross-sectional study was done among pregnant women in Ethiopia to evaluate knowledge, attitude, and practice of antenatal exercise. Among 349 women, adequate knowledge and a positive attitude toward exercise were found in 138 women (39.5%), and 193 women (55.3%) showed good practice toward antenatal exercise. Highly educated, governmental employees and those who had been advised to exercise before showed to have significantly greater knowledge, attitude, and practice of antenatal exercise [17]. This study aimed to evaluate the knowledge, beliefs, and practice of antenatal exercise and its association with the socio-demographic and maternal characteristics among pregnant women in Saudi Arabia in addition to identifying the most common barriers to exercising.

## Materials and methods

A cross-sectional online survey-based study was conducted in Saudi Arabia between March 2022 and May 2022. The target population was Saudi females who were pregnant or had been pregnant before. A self-administered questionnaire was used to collect the data. An informed consent form was provided to all participants before filling out the questionnaire. The questionnaire was provided electronically using Google Forms (Google, Mountain View, CA). The data were entered into Microsoft Excel (Microsoft Corporation, Redmond, WA) and subsequently uploaded and analyzed using the Statistical Package for the Social Sciences (SPSS) software (IBM Corp., Armonk, NY). Continuous variables were reported as mean ± SD, while categorical variables were reported using frequencies and percentages. All information was confidential and was only used for scientific research, and participation in this research was voluntary and optional with an informed consent form provided to all participants on the first page before filling out the questionnaire. This research was approved by the Biomedical Ethics Committee of Umm Al-Qura University (approval number: HAPO-02-K-012-2021-12-885).

Inclusion criteria and exclusion criteria

Saudi females (≥18 years old) who were pregnant or had been pregnant before were included in this study. Those who were not pregnant or had not been pregnant, women aged less than 18 years, non-Saudi females, and participants who did not fill out the whole questionnaire were excluded from the study.

Sampling technique and sample size calculation

OpenEpi® version 3.0 software was used to estimate our sample size, which is representative of Saudi Arabia's female population of 15 million. The representative sample size required was 385, with a margin error determined as 5% and a confidence level determined as 95%. We aimed to gather more than the estimated sample size to overcome potential exclusions. Non-probability convenience sampling techniques have been used.

Data collection instruments and procedures

The questionnaire was designed by the authors after an extensive review of the relevant literature. It was reviewed by experts in the field of obstetrics and gynecology to ensure clarity and simplicity and that all items in the questionnaire were relevant to the study purpose. The experts were contacted via emails and required to rate the relevance of each item in each section using a three-point Likert scale (1 = not relevant, 2 = somewhat relevant, and 3 = relevant) and to suggest other items that might not have been considered. The survey was designed in Arabic to make it easier for the population to read and understand. The final questionnaire was then pretested for content on 35 participants of different demographic characteristics, using its electronic version to ensure its coherence and wording. The participants who participated in the pretest were not included in the analysis. The developed questionnaire contained five sections that involved questions related to demographic information, beliefs, awareness, knowledge, and practice regarding antenatal exercises. Regarding the beliefs section, there were 10 statements with the options of agree, neutral, and disagree inquiring about common beliefs regarding exercise during pregnancy. Concerning awareness regarding antenatal exercise, the participants were asked to answer eight yes/no questions to measure their awareness level about the exercises practiced during pregnancy (e.g., abdominal muscle strengthening and Kegel exercises). Regarding the knowledge of the benefits of exercise during pregnancy, there were seven statements of evidence-based benefits of exercise during pregnancy, and the participants were asked to choose to answer the statement with yes, no, or I do not know, to assess their knowledge of these benefits. Regarding practicing exercise during pregnancy, the participants were asked to rank their activity level during pregnancy (always, very often, sometimes, rarely, and never).

The first page of the questionnaire was designated for informed consent. An electronic Google Forms survey was used and distributed on different social media platforms such as WhatsApp, Twitter, and Telegram. Using features of Google Forms such as “required to proceed” to make sure the study criteria would be fulfilled, the following question was provided at the beginning of the questionnaire: "Are you pregnant now or have you ever been pregnant?" If the answer was "yes," the participant would continue to go through questions in the questionnaire; however, if the answer was "no," the questionnaire form would be submitted directly.

Statistical analysis

The obtained data, including biographical knowledge and beliefs data, were arranged in Excel sheets. After data extraction, the data were revised, coded, and fed to the statistical software IBM SPSS version 23. Statistical analysis was done using two-tailed tests. A p-value less than 0.05 was statistically significant. Descriptive statistics were done based on the frequency distribution of all variables, including age, marital status, educational level, income, knowledge, and beliefs questions. The mean and standard error of the mean were calculated, and groups were compared using the Student’s t-test. Prevalence rates between groups were compared using the chi-square (x2) analysis using either 2 × 2 or 2 × 3 contingency tables. For all data comparisons, p < 0.05 was considered statistically significant.

## Results

Characteristics of the study participants

A total of 1662 respondents filled out the questionnaire. After applying the exclusion criteria, 1207 participants were included in the final analysis of the study. In regards to socio-demographic data, most of the participants (413, 34.2%) were within the age group of 26-35 years, 344 (28.5%) were within the age group of 18-25 years, 275 (22.8%) were between the age group of 36 and 45 years, and 175 (14.5%) were more than 45 years old. Moreover, 323 (26.8%) of the participants were living in the western region, 292 (24.2%) in the central region, 222 (18.4%) in the southern region, 189 (15.7%) in the northern region, and 181 (15%) in the eastern region. In regards to the marital status of the participants, 1070 (88.6%) were married, 79 (6.5%) were divorced, and 58 (4.8%) were widowed. University educational level was the most reported by 878 (72.7%) of the participants, followed by high school level, which was represented by 237 (19.6%) of the participants, intermediate educational level, which constitutes about 49 (4.1%) of the respondents, and 43 (3.6%) had elementary level education. Most of the respondents (489, 40.5%) were housewives, 441 (36.5%) were employed, 196 (16.2%) were students, and 81 (6.7%) were retired. The average household income (Saudi riyal (SAR)) was found to be from 5,000 to 10,000 SAR in 462 (38.3%) of the participants, from 10,000 to 20,000 SAR in 456 (37.8%) of the participants, and more than 20,000 SAR in about 157 (13%), and less than 5,000 SAR in 132 (10.9%) of the participants. Almost half of the participants (551, 45.7%) were found to be having one to three children and 325 (26.9%) were having four to six children. It was the first pregnancy in 243 (20.1%) of the participants. Of the participants, 87 (7.2%) had more than six children (grandmultiparous), and pregnancy was not completed (abortion) in one (0.1%) of the participants. Details of the socio-demographic characteristics of the participants are shown in Table 1.

**Table 1:** Socio-demographic characteristics of the participants (n = 1207)

Variable	Categories	Frequency	Percent
Age	18-25	344	28.5%
	26-35	413	34.2%
	36-45	275	22.8%
	>45	175	14.5%
Region	Southern	222	18.4%
	Northern	189	15.7%
	Central	292	24.2%
	Western	323	26.8%
	Eastern	181	15%
Marital status	Married	1070	88.6%
	Divorced	79	6.5%
	Widowed	58	4.8%
Educational level	Elementary	43	3.6%
	Intermediate	49	4.1%
	High school	237	19.6%
	University education	878	72.7%
Occupational status	Student	196	16.2%
	Employed	441	36.5%
	Housewife	489	40.5%
	Retired	81	6.7%
Average household income (Saudi riyal)	<5,000	132	10.9%
	5,000-10,000	462	38.3%
	10,000-20,000	456	37.8%
	>20,000	157	13%
No. of children	None (abortion)	1	0.1%
	This is my 1^st^ pregnancy	243	20.1%
	1-3	551	45.7%
	4-6	325	26.9%
	More than 6	87	7.2%

Beliefs, awareness, and knowledge regarding antenatal exercise

In regards to belief, awareness, and knowledge scores, out of a total score of 10, the mean beliefs score ± standard deviation was found to be 5.9 ± 2.1. Out of a total score of 8, the mean awareness score ± standard deviation was found to be 5.5 ± 2.2. Out of a total score of 7, the mean knowledge ± standard deviation was found to be 5.2 ± 2. The total score for beliefs, awareness, and knowledge was 25. The participants who correctly answered 50% of the questions (12.5, i.e., 12 out of 25 questions) were considered to have good knowledge and awareness. The mean score of the participants for beliefs, awareness, and knowledge ± standard deviation was found to be 16.5 ± 5.1. Regarding general knowledge and awareness levels, 1002 (83%) had a high level of knowledge and awareness, and 205 (17%) had a low level of knowledge and awareness.

Concerning beliefs regarding antenatal exercise, exercise during pregnancy is essential was agreed on by 853 (70.7%) of the participants, 73 (6%) disagreed with the same statement, and 281 (23.3%) were neutral. Exercising during pregnancy will help reduce and prevent complications during pregnancy was agreed on by 847 (70.2%) of the participants, 67 (5.6%) disagreed with the same statement, and 293 (24.3%) of the participants were neutral. Exercising regularly will facilitate the process of natural childbirth was agreed on by 987 (81.8%) of the participants, 36 (3%) disagreed with the same statement, and 184 (15.2%) of the participants were neutral. Doing exercises helps the recovery process after childbirth in a short period was agreed on by 932 (77.2%) of the participants, 60 (5%) disagreed with the same statement, and 215 (17.8%) of the participants were neutral. Exercising during pregnancy is safe for the baby and the mother was agreed on by 791 (65.5%) of the participants, 73 (6%) disagreed with the same statement, and 243 (28.4%) of the participants were neutral. Exercising during pregnancy does not suit our culture in Saudi Arabia was agreed on by 511 (42.3%) of the participants, 349 (28.9%) disagreed with the same statement, and 347 (28.7%) of the participants were neutral. Any pregnant mother can perform exercises without the advice and recommendations of healthcare professionals was agreed on by 337 (27.9%) of the participants, 672 (55.7%) disagreed with the same statement, and 198 (16.4%) of the participants were neutral. During pregnancy, the priority should be the improvement of nutrition and rest without physical exercise was agreed on by 554 (45.9%) of the participants, 265 (22%) disagreed with the same statement, and 388 (32.1%) participants were neutral. Performing daily household activities gives adequate physical exercise to pregnant women and they do not have to perform recommended exercises during pregnancy was agreed on by 492 (40.8%) of the participants, 357 (29.6%) disagreed with the same statement, and 358 (29.7%) of the participants were neutral. Exercises during pregnancy should be individually tailored to each pregnant woman was agreed on by 1022 (84.7%) of the participants, 47 (3.9%) disagreed with the same statement, and 138 (11.4%) of the participants were neutral. A promising finding in regards to awareness about antenatal exercise was found; about 1043 (86.4%) of the participants have previously heard about antenatal exercise. About 930 (77.1%) of the participants have previously heard about breathing exercises during pregnancy, and 793 (65.7%) of the participants have previously heard about back exercises during pregnancy. More than half of the participants (769, 63.7%) have heard about abdominal muscle strengthening exercises. Less than half of the participants (513, 42.5%) have heard about ankle-toe exercise during pregnancy. Moreover, more than half of the participants (796, 65.9%) have heard about aerobic exercises (such as swimming, walking, and cycling) during pregnancy, and about 910 (75.4%) have heard about yoga during pregnancy. Also, about 905 (75%) of the participants have heard about the Kegel exercise to strengthen the pelvic floor muscles during pregnancy.

Considering knowledge about exercise benefits during pregnancy, exercise reduces the risk of back pain during pregnancy was mentioned by 886 (73.4%) of the participants but 43 (3.6%) of the participants responded negatively and 278 (23%) participants mentioned do not know. Exercising during pregnancy prevents excessive weight gain in pregnancy was mentioned by 895 (74.2%) of the participants but 134 (11.1%) of the participants responded negatively and 178 (14.7%) of the participants mentioned do not know. Doing Kegel exercise during pregnancy strengthens pelvic floor muscles was mentioned by 856 (70.9%) of the participants but 92 (7.6%) of the participants responded negatively and 263 (21.8%) of the participants responded do not know. Exercise during pregnancy reduces the risk of developing diabetes in a pregnant woman was reported by 804 (66.6%) of the participants but 140 (11.6%) of the participants responded negatively and 263 (21.8%) of the participants responded do not know. Exercise during pregnancy increases energy was mentioned by 920 (76.2%) of the participants but 111 (9.2%) of the participants responded negatively and 176 (14.6%) of the participants responded do not know. Exercise during pregnancy will improve the ability to handle labor was stated by 950 (78.7%) of the participants but 96 (8%) of the participants responded negatively and 161 (11.1%) of the participants responded do not know. Exercise during pregnancy increases recovery after childbirth was reported by 916 (75.9%) of the participants but 109 (9%) of the participants responded negatively and 182 (15.1%) of the participants mentioned do not know. Items of beliefs, awareness, and knowledge regarding antenatal exercise are shown in Table 2.

**Table 2:** Beliefs, awareness, and knowledge regarding antenatal exercise

Beliefs regarding antenatal exercise	Agree	Disagree	Neutral
	n (%)	n (%)	n (%)
1. Exercise during pregnancy is essential	853 (70.7)	73 (6)	281 (23.3)
2. Exercising during pregnancy will help reduce and prevent complications during pregnancy	847 (70.2)	67 (5.6)	293 (24.3)
3. Exercising regularly will facilitate the process of natural childbirth	987 (81.8)	36 (3)	184 (15.2)
4. Doing exercises helps the recovery process after childbirth in a short period	932 (77.2)	60 (5)	215 (17.8)
5. Practice exercises during pregnancy is safe for the baby and the mother	791 (65.5)	73 (6)	343 (28.4)
6. Exercising during pregnancy does not suit our culture in Saudi Arabia	511 (42.3)	349 (28.9)	347 (28.7)
7. Any pregnant mother can perform exercises without the advice and recommendations of healthcare professionals	337 (27.9)	672 (55.7)	198 (16.4)
8. During pregnancy, the priority should be the improvement of nutrition and rest without physical exercise	554 (45.9)	265 (22)	388 (32.1)
9. Performing daily household activities gives adequate physical exercise to pregnant women and they do not have to perform recommended exercises during pregnancy	492 (40.8)	357 (29.6)	358 (29.7)
10. Exercises during pregnancy should be individually tailored to each pregnant woman	1022 (84.7)	47 (3.9)	138 (11.4)
Awareness regarding antenatal exercise		Yes	No
1. Have you ever heard about ante-natal exercise?		1043 (86.4)	164 (13.6)
2. Have you ever heard about breathing exercises during pregnancy?		930 (77.1)	277 (22.9)
3. Have you ever heard about back exercise during pregnancy?		793 (65.7)	414 (34.3)
4. Do you know abdominal muscle strengthening exercises during pregnancy?		769 (63.7)	438 (36.3)
5. Have you ever heard about ankle-toe exercise during pregnancy?		513 (42.5)	694 (57.5)
6. Have you heard about aerobic exercise (such as swimming, walking, and cycling) during pregnancy?		796 (65.9)	411 (34.1)
7. Have you ever heard about yoga during pregnancy?		910 (75.4)	297 (24.6)
8. Have you heard of Kegel exercises to strengthen the pelvic floor muscles during pregnancy?		905 (75)	302 (25)
Knowledge of benefits of exercise during pregnancy	Yes	No	I don’t know
1. Exercise reduces the risk of back pain during pregnancy	886 (73.4)	43 (3.6)	278 (23)
2. Exercise during pregnancy to prevent excessive weight gain in pregnancy	895 (74.2)	134 (11.1)	178 (14.7)
3. Doing Kegel exercises during pregnancy strengthens pelvic floor muscle	856 (70.9)	92 (7.6)	259 (21.5)
4. Exercising during pregnancy reduces the risk of developing diabetes in pregnant women	804 (66.6)	140 (11.6)	263 (21.8)
5. Exercise during pregnancy increases energy	920 (76.2)	111 (9.2)	176 (14.6)
6. Exercising during pregnancy will improve the ability to handle labor and delivery	950 (78.7)	96 (8)	161 (13.3)
7. Exercising during pregnancy increases recovery after childbirth	916 (75.9)	109 (9)	182 (15.1)

**Figure 1:**
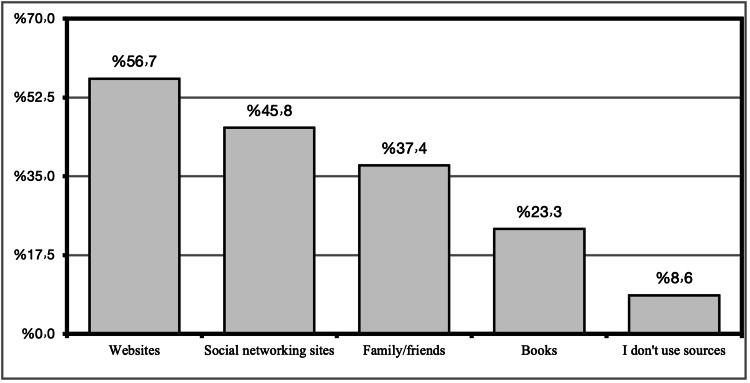
Sources of information on prenatal exercise

The main source of information on prenatal exercise was found to be websites, as reported by 56.7% of the participants, and social media was the source of information on prenatal exercise in about 45.8% of respondents, whereas in 37.4%, the source of information was family or friends, in 23.3%, the source of information was books, and 8.6% did not use any source. Figure 1 illustrates the sources of information on prenatal exercise.

Practicing exercise during pregnancy

**Figure 2:**
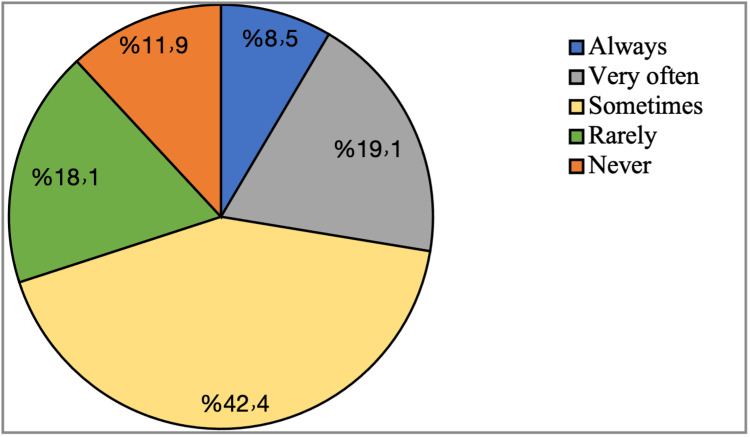
How often the participants exercise during pregnancy

About 42.4% of the participants were sometimes exercising during pregnancy, 19.1% of the participants exercise very often, 18.1% of the participants rarely exercised, 11.9% of the participants never exercised, and 8.5% always exercise. Figure 2 shows how often participants exercise during pregnancy.

**Figure 3:**
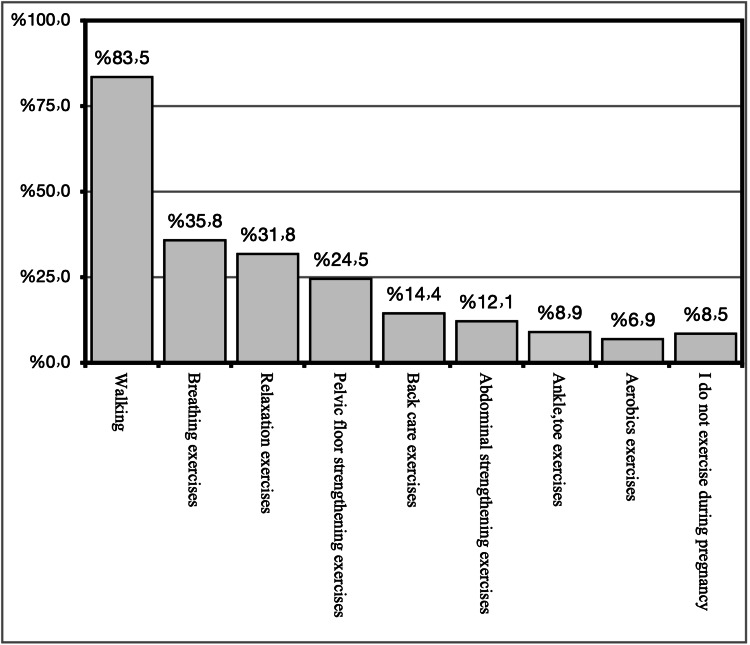
The type of antenatal exercise participants are practicing

The main type of antenatal exercise was found to be walking, as mentioned by 83.5% of the participants, breathing exercises were mentioned by 35.8% of the participants, relaxation exercises were practiced by 31.8% of the participants, pelvic floor strength exercises were reported by 24.5% of the participants, 14.4% practiced back care exercises, abdominal strengthening exercises were practiced by 12.1% of the participants, ankle and toe exercises were practiced by 8.9% of the participants, aerobic exercises were practiced by 6.9% of pregnant women, and 8.5% of the participants do not exercise during pregnancy. Figure 3 illustrates the type of antenatal exercise participants were practicing.

**Figure 4:**
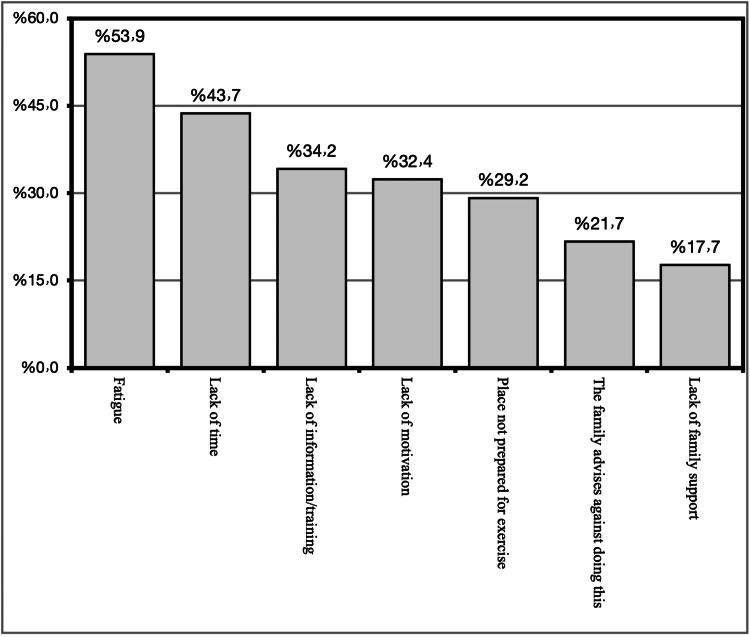
The most common barriers to practicing antenatal exercise

The most commonly reported barrier to practicing antenatal exercises was found to be fatigue, as reported by 53.9% of the participants, lack of time was the barrier to antenatal exercise in 34.2% of the participants, and lack of information and training was the barrier for antenatal exercise in 34.2% of the participants. Lack of motivation was the barrier in about 32.4% of the participants, in 29.2% of the participants, the barrier was that the place was not prepared for exercise, family advice against training was the barrier in about 21.7% of the participants, and lack of family support was the barrier in 17.7% of the participants. Figure 4 shows the most common barriers to practicing antenatal exercise.

Factors associated with the level of beliefs, awareness, and knowledge regarding antenatal exercise

Age was found to be significantly associated with the level of beliefs, awareness, and knowledge regarding antenatal exercise (p = 0.001), with the age group of 18-25 years found to be more aware and knowledgeable than the other age groups. The region was not found to be significantly associated with the level of beliefs, awareness, and knowledge regarding antenatal exercise (p = 0.640). A statistically significant association was found between marital status and level of beliefs, awareness, and knowledge regarding antenatal exercise (p < 0.001), with married participants tending to be having a higher level of knowledge and awareness compared to other groups. Educational level was found to be significantly associated with the level of beliefs, awareness, and knowledge regarding antenatal exercise (p = 0.002), with the university educational level having higher knowledge and awareness compared to other educational levels. Occupation and level of beliefs, awareness, and knowledge regarding antenatal exercise were found to be significantly associated (p =< 0.001) with student participants more frequently having higher knowledge and awareness levels. No significant association was found between average household income and level of beliefs, awareness, and knowledge regarding antenatal exercise (p = 0.298). The number of children was found to be significantly associated with the level of beliefs, awareness, and knowledge regarding antenatal exercise (p < 0.001), with participants having one to three children being more aware and knowledgeable than others. Table 3 illustrates the association between socio-demographic data and the level of beliefs, awareness, and knowledge regarding antenatal exercise.

**Table 3:** Association between socio-demographic data and level of beliefs, awareness, and knowledge regarding antenatal exercise

Variable	Categories	Level of beliefs, awareness, and knowledge		P-value
		High	Low	
Age	18-25	304 (88.4)	40 (11.6)	0.001
	26-35	341 (82.6)	72 (17.4)	
	36-45	226 (82.2)	49 (17.8)	
	>45	131 (74.9)	44 (25.1)	
Region	Southern	188 (84.7)	34 (15.3)	0.640
	Northern	150 (79.4)	39 (20.6)	
	Central	243 (83.2)	49 (16.8)	
	Western	268 (83)	55 (17)	
	Eastern	153 (84.5)	28 (15.5)	
Marital status	Married	911 (85.1)	159 (14.9)	<0.001
	Divorced	60 (75.9)	19 (24.1)	
	Widowed	31 (53.4)	27 (46.6)	
Educational level	Elementary	36 (83.7)	7 (16.3)	0.002
	Intermediate	36 (73.5)	13 (26.5)	
	High school	180 (75.9)	57 (24.1)	
	University education	750 (85.4)	128 (14.6)	
Occupational status	Student	174 (88.8)	22 (11.2)	<0.001
	Employed	371 (84.1)	70 (15.9)	
	Housewife	404 (82.6)	85 (17.4)	
	Retired	53 (65.4)	28 (34.6)	
Average household income (Saudi riyal)	<5,000	104 (78.8)	28 (21.2)	0.298
	5,000-10,000	394 (85.3)	68 (14.7)	
	10,000-20,000	374 (82)	82 (18)	
	>20,000	130 (82.8)	27 (17.2)	
No. of children	None (abortion)	1 (100)	0 (0)	<0.001
	This is my 1^st^ pregnancy	203 (83.5)	40 (16.5)	
	1-3	479 (86.9)	72 (13.1)	
	4-6	262 (80.6)	63 (19.4)	
	More than 6	57 (65.5)	30 (34.5)	

## Discussion

Exercise was found to be beneficial during pregnancy with positive effects on both mother and her baby and it was also found to be associated with improved pregnancy outcomes [7-10]. Assessing knowledge and practice about exercise during pregnancy will shed the light on the limitations and barriers against exercise as beneficial activity, and also it will assist in the determination of the proper way of intervention [18]. The current study aimed to assess females’ knowledge, beliefs, and practices toward exercise during pregnancy in Saudi Arabia and its associated factors and to identify the relevant barriers to exercising.

Most of the participants (34.2%) were within the age group of 26-35 years. The vast majority (88.6%) of respondents were married. University educational level was the most reported, as more than two-thirds (72.7%) of the participants had university-level education. The total score for beliefs, awareness, and knowledge was 25. The mean score for beliefs, awareness, and knowledge was found to be 16.5, and this was similar to a parallel study in which out of a score of 20, knowledge and awareness scores were found to be 9.85 ± 2.7 SD [17].

Regarding general knowledge and awareness levels, the majority (83%) of the participants had a high level of knowledge and awareness, and about 17% had low knowledge and awareness level. This was found to be consistent with another study in which the vast majority of the participants were with good knowledge level about exercise during pregnancy [19]. Exercise during pregnancy is essential as agreed by more than two-thirds (70.7%) of the participants. Exercising during pregnancy will help reduce and prevent complications during pregnancy was mentioned by more than two-thirds (70.2%) of the participants. Practicing exercise during pregnancy is safe for the baby and the mother was reported by nearly two-thirds (65.5%) of the participants. This was similar to findings in another study in which the same beneficial effects were mentioned [20]. Exercising during pregnancy does not suit our culture in Saudi Arabia was agreed on by more than one-third (42.3%) of the participants, and this was found to agree with the findings elaborated in the congruent study that also found that antenatal exercise is not part of Saudi Arabian culture [21]. Any pregnant mother can perform exercises without the advice and recommendations of healthcare professionals was reported by less than one-third (27.9%) of the participants. This was found to be contradictory to a study that reported that healthcare professionals' advice is essential before starting antenatal exercise [22].

Considering knowledge about exercise benefits during pregnancy, exercise reduces the risk of back pain during pregnancy was reported by more than two-thirds (73.4%). Exercise during pregnancy prevents excessive weight gain in pregnancy was mentioned by more than 74.2% of the participants. Exercise during pregnancy increases recovery after childbirth was reported by most (75.9%) of the participants. All the previously mentioned exercise benefits were also reported in another parallel study in which improved pregnancy outcome was also improved with exercise [11].

Sources of information on prenatal exercise were found to be websites, as reported by 56.7% of the participants. Social media was the source of information on prenatal exercise in about 45.8% of respondents, whereas in 37.4% of the participants, the source of information was family or friends. These results were found to be consistent with the findings stated in a parallel study in which the most reported sources of information about exercise were the internet and media [23].

We found that about 42.4% of the participants were sometimes exercising during pregnancy, and this finding was promising and higher than what was reported in a study in which only 10% of the participants were exercising [24]. In our study, the most reported type of antenatal exercise was found to be walking, as mentioned by 83.5% of the participants, and this was comparable with the congruent study in which the most recommended type of exercise was aerobic exercises [25].

As expected, the most commonly reported barrier to practicing antenatal exercises was found to be fatigue, as reported by 53.9% of the participants, followed by lack of time. The same findings were reported in a similar study in which fatigue and lack of time were the most reported barriers to exercise during pregnancy [26].

Age was found to be significantly associated with the level of beliefs, awareness, and knowledge regarding antenatal exercise, with the age group of 18-25 years found to be more aware and knowledgeable than the other age groups. A statistically significant association was shown between marital status and level of beliefs, awareness, and knowledge regarding antenatal exercise, with married participants tending to be having a higher level of knowledge and awareness compared to other groups. Similar findings were reported in a study in which married female participants were more aware and tend to practice antenatal exercise more than unmarried participants [27]. Educational level was found to be significantly associated with the level of beliefs, awareness, and knowledge regarding antenatal exercise, with participants with university-level education having higher knowledge and awareness compared to other educational levels. This was found to be consistent with another study in which education and age were reported as the most commonly associated with knowledge and awareness [28]. Occupation and level of beliefs, awareness, and knowledge regarding antenatal exercise were found to be significantly associated, with student participants more frequently having higher awareness and knowledge levels. This was found to be contradictory to the findings reported in a parallel study in which occupation was only found to be significantly associated with attitude and beliefs but not found to be associated with knowledge and awareness about antenatal exercise [29]. In our study, we found that the number of children was found to be significantly associated with the level of beliefs, awareness, and knowledge regarding antenatal exercise with participants having one to three children being more aware and knowledgeable than others. This was found to be in contradiction to another local study in which a low number of children was found to be associated with low knowledge and awareness about exercise during pregnancy [30]. An area of strength in this study is that the questionnaire employed achieved a high response rate. Another strength is that the sample was collected from different regions of Saudi Arabia, which allows the generalization of the findings. However, the study was limited by being a questionnaire-based study, which inherently has the risk of recall, interviewer, and response bias. It is crucial to understand the barriers and beliefs that influence physical activity during pregnancy. As a result, prenatal program care should be restructured and particular measures developed to overcome these barriers and promote physical activity among pregnant women. Future studies are recommended to understand the factors associated with exercising during pregnancy to implement effective strategies for proper antenatal care.

## Conclusions

We found good general knowledge and awareness levels regarding exercise during pregnancy; however, beliefs about exercise during pregnancy were below average. Fatigue and lack of time were the most reported barriers. Age and educational level were significantly associated with knowledge and awareness about exercise during pregnancy. Exercise education programs are recommended to motivate pregnant women and to change common misbeliefs regarding antenatal exercise.
